# The secreted tyrosine phosphatase PtpA promotes *Staphylococcus aureus* survival in RAW 264.7 macrophages through decrease of the SUMOylation host response

**DOI:** 10.1128/spectrum.02813-23

**Published:** 2023-10-11

**Authors:** Nadhuma Youssouf, Marianne Martin, Markus Bischoff, Philippe Soubeyran, Laila Gannoun-Zaki, Virginie Molle

**Affiliations:** 1 VBIC, INSERM U1047, Université de Montpellier, Montpellier, France; 2 Institute for Medical Microbiology and Hygiene, Saarland University, Homburg, Saarland, Germany; 3 Centre de Recherche en Cancérologie de Marseille (CRCM), INSERM U1068, CNRS UMR, Aix-Marseille, Université and Institut Paoli-Calmettes, Parc Scientifique et Technologique de Luminy, Marseille, France; Ludwig-Maximilians-Universitat Munchen Pettenkofer Institute, München, Germany

**Keywords:** *Staphylococcus aureus*, SUMOylation, secreted phosphatase, PtpA, macrophage survival

## Abstract

*Staphylococcus aureus* is a human pathogen that is extremely adaptable and is the cause of a variety of nosocomial and community-acquired infectious diseases. During infection, *S. aureus* is able to affect the host cell in many ways to enable its own multiplication, spread, and evasion of the host immune defense. One of the mechanisms utilized by *S. aureus* to survive is to inhibit the SUMOylation of host proteins in order to increase its intracellular survival and persistence. Here, we show that the reduction in the levels of cellular SUMO-conjugated proteins in *S. aureus* strain Newman-infected RAW 264.7 cells is associated with the *S. aureus* secreted protein tyrosine phosphatase PtpA, which results in a reduction of ubiquitin-conjugating enzyme 9 (Ubc9) protein level, the critical enzyme of the SUMOylation modification. In addition, we demonstrate that the amino acid residue D120, which is essential for PtpA phosphatase activity, is required for this reduction. This study shows for the first time that *S. aureus* strain Newman impedes via PtpA the host SUMOylation response, which contributes to promoting the persistence of *S. aureus* within the host.

**IMPORTANCE:**

*Staphylococcus aureus* uses numerous strategies to survive and persist in the intracellular environment of professional phagocytes, including modulation of the SUMOylation process. This study aims to understand how *S. aureus* alters host SUMOylation to enhance its intracellular survival in professional phagocytes. Our results indicate that *S. aureus* strain Newman utilizes PtpA-driven phosphorylation to decrease the amount of SUMOylated proteins in murine macrophages to facilitate its survival in this immune cell type.

## INTRODUCTION

Pathogenic bacteria often affect the host cell physiology during infection to enable their own multiplication, spread, and evasion of the host immune defense (1). Post-translational modifications (PTMs), which are essential for controlling the location, activity, and interaction of proteins are often involved in these host-interaction processes upon infection (2). PTMs comprise phosphorylation, acetylation, and methylation, as well as the incorporation of small polypeptides like ubiquitin or ubiquitin-related proteins such as the small ubiquitin-like modifiers (SUMOs). It is known that a number of pathogens highjack PTMs for their own benefit; however, it is only established that a small number of pathogenic microorganisms may interfere with the SUMOylation pathway (2 – 6). SUMOylation is a type of reversible post-translational modification that occurs in eukaryotic cells. In this regulation, a SUMO protein is covalently bound to its target proteins (7), thereby influencing cellular functions such as DNA replication, the transcription of genetic information, processing of RNA, and cell signaling (8, 9). Only very recently, researchers started looking into the strategies utilized by pathogenic bacteria to alter SUMOylation of host proteins, and our understanding of these processes is still limited (10). In a recent study, we were able to show that the human pathogen *Staphylococcus aureus* inhibits the SUMOylation of host proteins in order to increase its intracellular survival and persistence (11). Additionally, a correlation between the decreased degree of SUMOylation and the reduction in the amount of the SUMO-conjugating enzyme Ubc9 was observed. Moreover, artificially increased SUMOylation in macrophages was shown to reduce the intracellular proliferation of bacteria, whereas treatment with the SUMOylation inhibitor ML-792 led to an increased bacterial survival within this immune cell type (11). Interestingly, human pathogens such as *Listeria monocytogenes* (2) and *Yersinia pestis* (12), or the plant pathogen *Xanthomonas euvesicatoria* (13) have been shown to release effectors that are able to elicit a general deSUMOylation. *S. aureus* is an opportunistic human pathogen that is extremely versatile and the cause of a variety of nosocomial and community-acquired diseases (14, 15). Pathogenicity of this bacterium is largely attributed to its reservoir of virulence factors and regulatory elements (16, 17). The bacterium is able to invade a variety of non-professional and professional phagocytic cells, where it may persist for many days (18 – 20). PtpA is a low-molecular-weight protein tyrosine phosphatase that is secreted by *S. aureus*. We have previously shown that PtpA is released during growth and macrophage infection, and that deletion of *ptpA* reduces *S. aureus* intramacrophage survival and infectivity (21). In this study, we show that a reduction in the levels of cellular SUMO-conjugated proteins is associated with PtpA, which causes a reduction of the Ubc9 level, the essential enzyme of the SUMOylation modification machinery. In addition, we demonstrate here that the phosphatase activity is required for the PtpA-dependent reduction in SUMOylation.

## RESULTS

### *S. aureus* PtpA phosphatase activity is required for survival in murine macrophages

**Fig 1:**
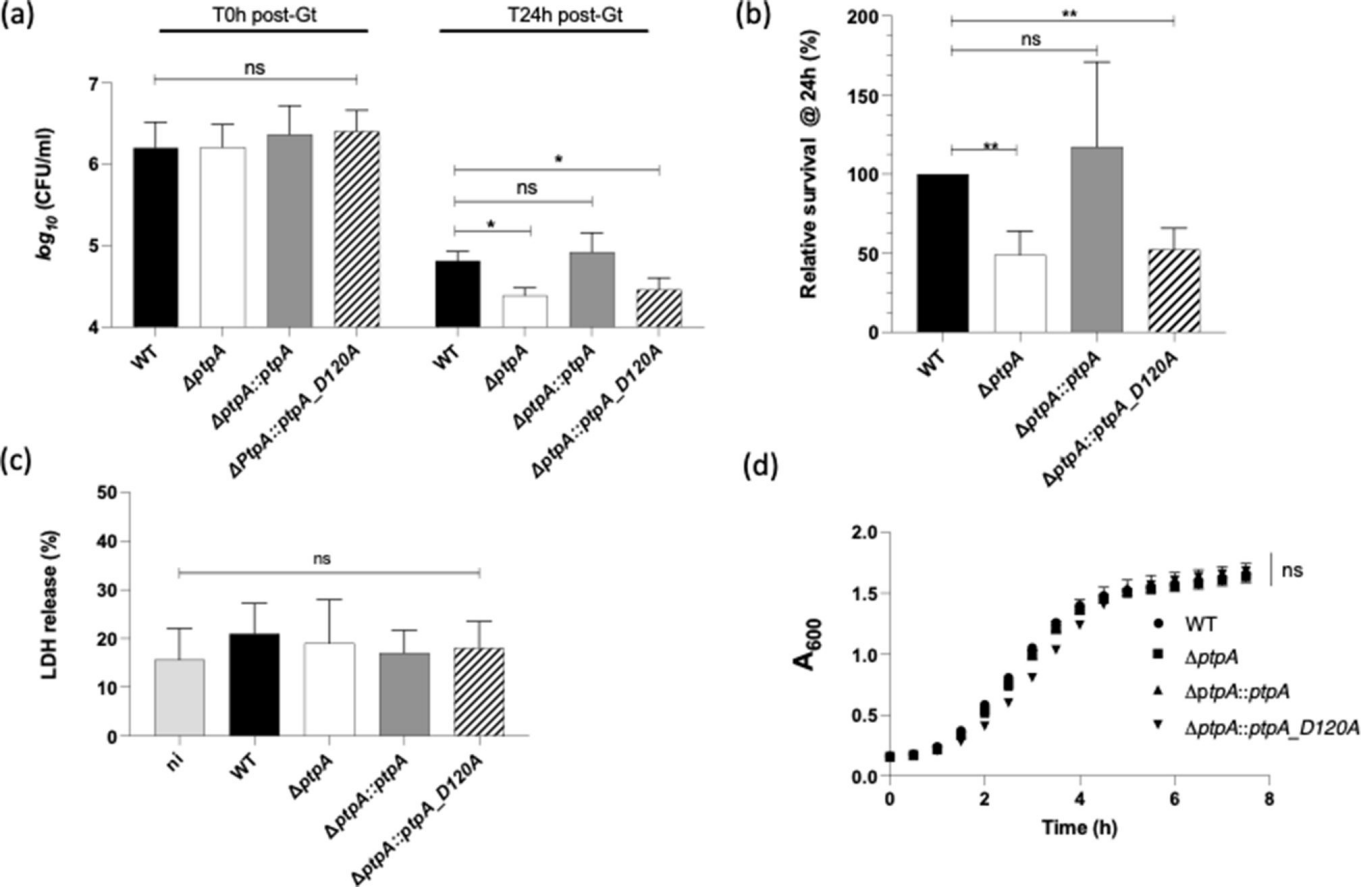
Impact of PtpA on survival and cytotoxicity of *S. aureus* in macrophages. (a, b) Long-term survival of *S. aureus* in infected macrophages. Cells of *S. aureus* strains Newman (WT; black bar), Newman Δ*ptpA* (white bar), Newman Δ*ptpA::ptpA* (gray bar), and Newman Δ*ptpA::ptpA_D120A* (hatched bar) were used to infect RAW 264.7 macrophages at a multiplicity of infection (MOI) of 20 and co-incubated for 1 h at 37°C before extracellularly remaining bacterial cells were killed by gentamicin/lysostaphin treatment. Bacteria were counted on plates after macrophage lysis with Triton X100 (0.1%) at 24-hr pGt and represented as CFU enumeration (a) or survival rates expressed in relation to the number of intracellular bacterial cells counted just after gentamicin administration and normalized to the survival rates seen with WT-infected macrophages at 24-h pGt (b). Data show means and standard deviations (SDs) (*n* = 4). *P < 0.05, **P < 0.01 and ns: not significant (Mann-Whitney *U* test). (c) LDH release was evaluated using the CyQUANT test kit after macrophage cells were infected with bacteria at an MOI of 20 for 24 h. The cells were seeded in a 96-well plate for 25 min and the LDH release was determined. The data on four biological replicates are shown relative to the 100% positive control corresponding to total cell lysis with Triton X100 (1%). Non-infected RAW 264.7 cells served as negative control (ni). ns: not significant (Mann-Whitney *U* test). (d) *In vitro* growth kinetics. Growth of *S. aureus* strains Newman, Newman Δ*ptpA*, Newman Δ*ptpA::ptpA*, and Newman Δ*ptpA::ptpA_D120A* were performed in TSB at 37°C and 110 rpm in 96-well plates using a microplate reader (Tecan, Lyon, France). Data represent the mean *A*_600_ readings at the time points indicated (*n* = 3). ns: not significant (one-way analysis of variance test).

We recently showed that PtpA enhances the intracellular survival of *S. aureus* in murine macrophages (21), but it is unknown yet, whether the phosphatase activity of PtpA is necessary for this intramacrophage survival capacity of *S. aureus*. In order to test this, we created a PtpA phosphatase deficient mutant in *S. aureus* strain Newman (Newman Δ*ptpA::ptpA*_*D120A*), based on earlier findings showing that residue D120 in the catalytic loop of PtpA is necessary for its phosphatase activity (22). Next, we determined how cells of the *S. aureus* strains Newman wild type (WT), Newman Δ*ptpA*, Newman Δ*ptpA::ptpA*, and Newman Δ*ptpA::ptpA*_*D120A* survived inside cells of the murine macrophage cell line RAW 264.7. Intracellular CFU counts at T0 were similar between all strains used to infect the RAW 264.7 cells, thus ruling out a phagocytosis defect of the mutant (Fig. 1a). However, at 24-h post-gentamicin treatment (pGt), intracellular bacteria loads decreased significantly for Newman Δ*ptpA::ptpA*_*D120A*, when compared to the WT (Fig. 1b). In line with our previous findings (21), survival rates of the Δ*ptpA* mutant also dropped to around 50% of the survival rates seen in macrophages that had been infected with the WT and the *cis*-complemented Newman Δ*ptpA::ptpA* strain, respectively (Fig. 1b). These findings confirmed, on the one hand, the important role PtpA plays for the capacity of *S. aureus* to persist within murine macrophages and demonstrated, on the other hand, that the phosphatase activity of PtpA is required for the intracellular survival of *S. aureus* strain Newman in murine macrophages. To exclude that the decreased numbers of Newman ΔptpA and Newman Δ*ptpA::ptpA_D120A* cells seen in infected RAW 264.7 cells at 24-h pGt might be due to alterations in bacterial cytotoxicity elicited by these strains during the intracellular passage, we next used the lactate dehydrogenase (LDH) assay to determine the damage rates of the infected macrophage cells. The LDH test is a classic assay for identifying cytotoxicity by evaluating the level of damage to the cellular plasma membrane via the amount of LDH enzyme that is released into the culture media (23). Notably, infection with all four *S*. *aureus* strains resulted in LDH release rates in the culture supernatants over time that were comparable to the LDH release rates seen with uninfected RAW 264.7 macrophages (Fig. 1c). This finding indicates that *S. aureus* strain Newman and its *ptpA* derivatives are able to survive intracellularly in macrophages for extended periods of time without inducing clear cytotoxicity. This observation also ruled out that the reduced bacterial cell numbers seen in Newman Δ*ptpA* and Newman Δ*ptpA::ptpA_D120A* infected RAW 264.7 cells at 24-hr pGt were due to elevated cytotoxicity of the internalized bacterial cells. Elevated cytotoxicity would have led to an enhanced killing of the infected macrophages and a subsequent release of bacterial cells into the extracellular milieu in which large proportions of the released cell population would have been killed by the lysostaphin present in the cell culture medium. To test whether complementation of the Newman Δ*ptpA* mutant with *ptpA*_D120A might have an effect on the growth behavior, we also studied its growth in tryptic soy broth (TSB) at 37°C and 225 rpm over time (Fig. 1d). The *in vitro* growth curves obtained with all four Newman derivatives yielded rather comparable growth kinetics, except for a non-significant reduced growth of the Δ*ptpA::ptpA_D120A* mutant during the exponential growth phase (i.e., 2–4 h).

### *S. aureus* PtpA phosphatase is involved in the decrease of host SUMOylation upon infection

**Fig 2:**
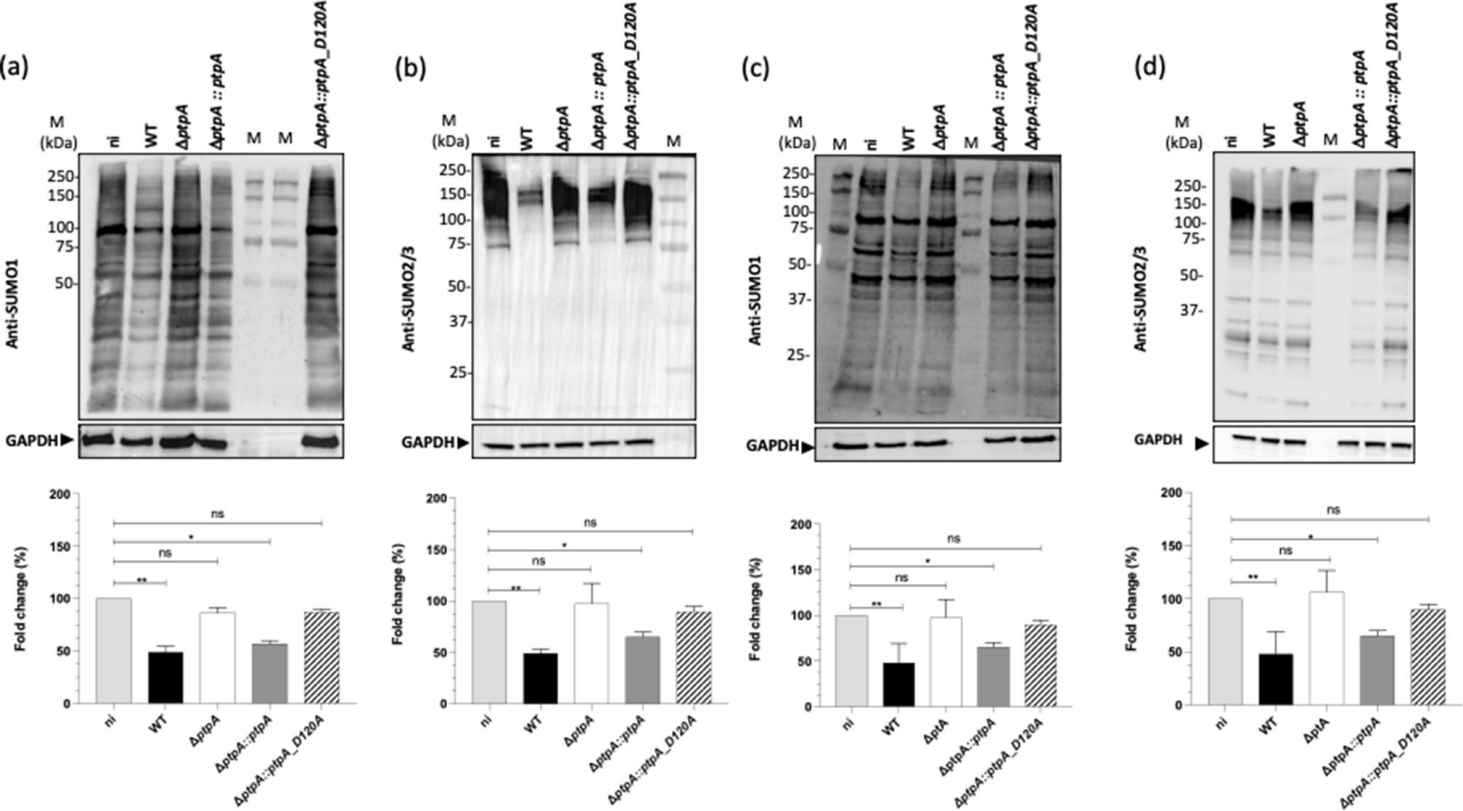
PtpA decreases SUMO-conjugated proteins in *S. aureus*-infected macrophages. Immunoblot analyses of the levels of SUMO1 (a), SUMO2/3 (b), and glyceraldehyde-3-phosphate dehydrogenase (GAPDH) in the lysates of *S. aureus*-infected macrophages at 24-h pGt. Immunoblot analyses of the levels of SUMO1 (c), SUMO2/3 (d), and GAPDH in the lysates of *S. aureus*-infected macrophages at 24-h pGt using different MOI (MOI 10 for WT strain and MOI 20 for Δ*ptpA*, Δ*ptpA::ptpA*, and Δ*ptpA::ptpA_D120A*). Using Image Lab software (ChemiDoc), SUMO1 and SUMO2/3 smears were quantified from four different experiments and normalized to the GAPDH signals (lower panels). The fold change charts display the proportion of SUMOylated proteins recovered from infected cells in comparison to the quantity of SUMOylated proteins in non-infected (ni) control macrophages. The data represented are the mean ± SD of four biological experiments. **P < 0.01; *P < 0.05; and ns: not significant (Kruskal-Wallis test followed by Dunn’s *post hoc* test; only differences between non-infected and infected cells are shown).

Given the findings that a ptpA deletion or D120 mutation reduced the intramacrophage survival of *S. aureus*, and based on our recent observations showing that *S. aureus* inhibits the SUMOylation of host proteins in order to increase its intracellular survival and persistence in macrophages (11), we wondered whether PtpA might be involved in the interference with the host SUMOylation response of RAW 264.7 cells to *S. aureus* infection. In order to test this hypothesis, we analyzed the amounts of SUMO1- and SUMO2/3-conjugated proteins in uninfected RAW 264.7 cells and infected RAW 264.7 cells at 24-h pGt. First, in comparison to non-infected cells, macrophages that were infected with *S. aureus* Newman showed a significant and specific decrease in the amount of SUMO1 (Fig. 2a) and SUMO2/3 (Fig. 2b) modified proteins, in accordance with our previous observations (11). On the other hand, the global pattern of SUMO-conjugated proteins was rather comparable to that of non-infected RAW 264.7 cells (ni) when RAW 264.7 cells were infected with Newman Δ*ptpA* and Δ*ptpA::ptpA_D120A* cells, respectively (Fig. 2). In contrast, decreased SUMOylation profiles were again observed in RAW 264.7 cells infected with the *cis*-complemented Newman Δ*ptpA::ptpA* derivative (Fig. 2). These data strongly suggest that PtpA phosphatase activity plays a major role in the decrease in SUMOylation observed in RAW 264.7 cells infected with *S. aureus*. As demonstrated in our previous study, *S. aureus* Newman survival is decreased at 5-h post-infection (11). However, despite the high amount of intracellular bacteria after 5 h of infection (around 5.75 × 10^5^ CFU/mL or 12% survival rate), no reduction in the amount of SUMOylated proteins in WT-infected macrophages in comparison with uninfected macrophages was observed. On the other hand, after 24 h of infection, the intracellular survival of strain Newman was further reduced by 1log_10_ (around 2 × 10^4^ CFU/mL or 3% survival rate) and a decrease in the amount of SUMOylated proteins in these infected macrophages was observed (11). In addition, we can exclude the possibility that differences in SUMOylation profile between macrophages infected with *S. aureus* strains Newman or Newman Δ*ptpA* are due to the reduced number of intracellular bacteria. This demonstration was performed by reducing the MOI of the wild-type strain (MOI 10) to half of the one used for the mutant (MOI 20) as the survival default is about 50% between strains (Fig. 2c and d). These observations allow us to exclude the hypothesis that the absence of SUMOylation reduction by *S. aureus* Δ*ptpA* is linked to a low quantity of intracellular bacteria after 24 h of infection.

### *S. aureus* PtpA reduces Ubc9 level in a transcriptional-independent manner

**Fig 3:**
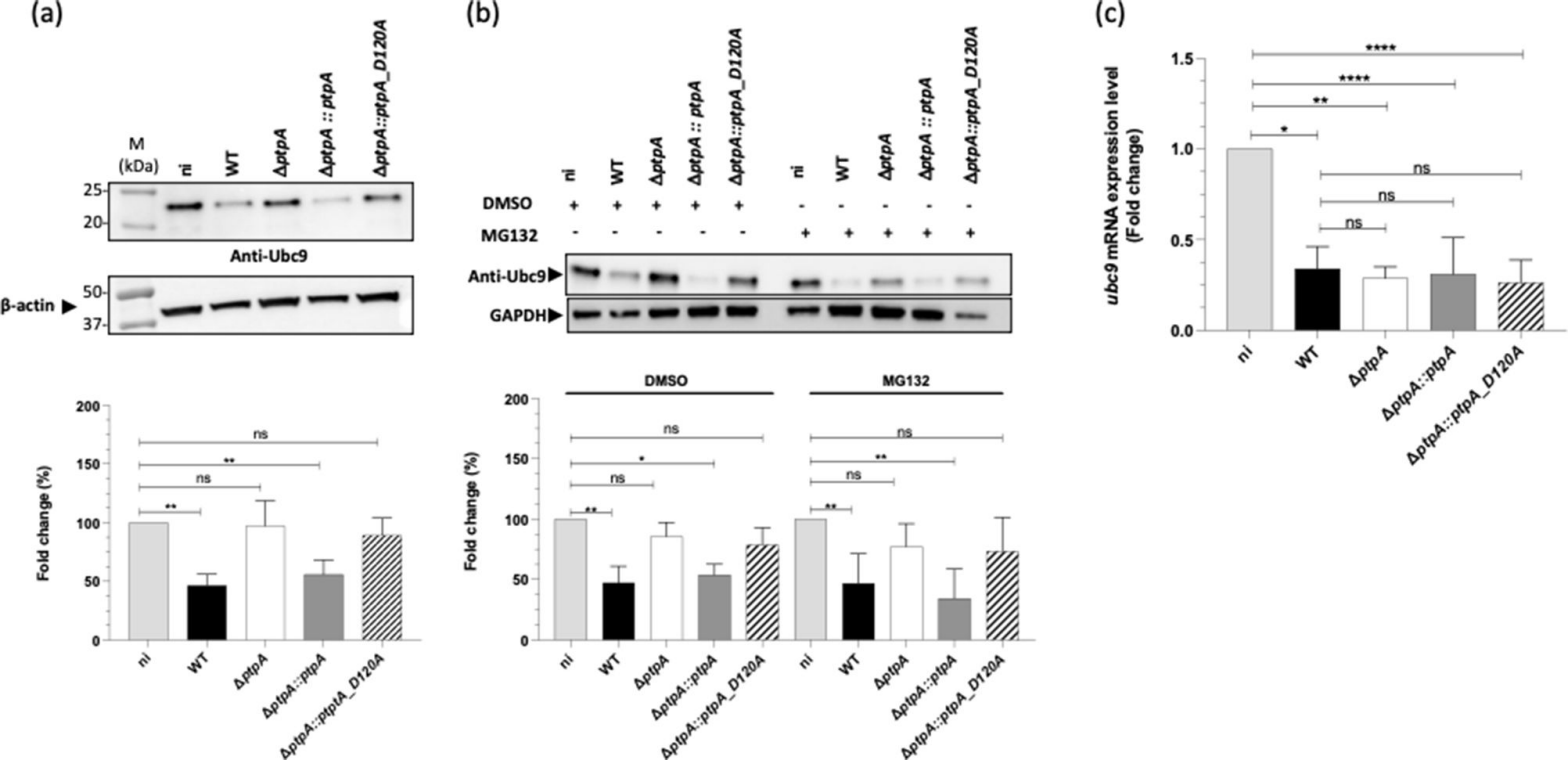
*S. aureus* PtpA reduces Ubc9 level but not *ubc9* transcription. Immunoblot analysis of Ubc9 levels in lysates of macrophages not treated (a), or treated with MG132 for 3 h prior to infection (b), and infected with *S. aureus* strains for 24- h post-gentamicin treatment. ni, non-infected control cells. Ubc9 bands were quantified from four independent experiments and normalized to β-actin or GAPDH levels. The graph represents fold changes compared to non-infected cells (bottom panel). *P < 0.05 and ns: not significant (Kruskal-Wallis test followed by Dunn’s *post hoc* test). (c) The influence of PtpA on the transcription of *ubc9*. qRT-PCR was used to perform quantitative assessments of the *ubc9* transcript in *S. aureus* cells that had been cultured for 24 h after being treated with gentamicin. Quantification of transcription rates was done in relation to the transcription of β-*act* (in copies per copy of *β-actin*), which was used as the standard, and normalized to the transcript rates seen with the uninfected controls. The data are provided as mean + SD of four separate biological experiments. *P < 0.05, **P < 0.01, ****P < 0.0001 and ns: not significant (Kruskal-Wallis test followed by Dunn’s *post hoc* test).

After demonstrating that PtpA is a critical factor for *S. aureus* intramacrophage survival and that it is responsible for the reduction of SUMOylation in this immune cell type, we wondered how PtpA might accomplish this regulation. One potential effector molecule might be the ubiquitin-conjugating enzyme 9 (Ubc9), which is the only E2 conjugating enzyme of the SUMOylation machinery required for the SUMOylation to occur (9). Therefore, we started by measuring the amount of the Ubc9 enzyme present in macrophages infected with our *S. aureus* Newman strain set. We observed that the Ubc9 protein level in macrophages infected with *S. aureus* Newman or the *ptpA*-complemented strain decreased by approximately 50% at 24-h pGt when compared to uninfected cells (Fig. 3a). In contrast, no clear reductions in Ubc9 signals were observed in macrophages infected with the Δ*ptpA* derivative and the PtpA phosphatase-inactive *ptpA_D120A* derivative, respectively (Fig. 3a). Next, we utilized the proteasome inhibitor MG132 in order to evaluate whether the proteasome had a role in Ubc9 reduction. These experiments revealed that inhibition of proteasome activity by MG132 had no impact on the amount of Ubc9 (Fig. 3b), which continued to drop in macrophages infected with *S. aureus* strains expressing a WT PtpA. In addition, quantitative reverse transcription (qRT)-PCR was used to examine the levels of *ubc9* expression. Here, we found that *S. aureus* infection indeed affected the expression of the Ubc9 enzyme at the mRNA level (Fig. 3c), however, in a rather PtpA-independent manner (Fig. 3c). Taken together, these findings suggest that PtpA does have an effect on the quantity of Ubc9, though, without affecting the proteasome and the transcriptional pathway, respectively.

### Modulating the level of SUMOylation confirms the role of PtpA in promoting intracellular survival of *S. aureus* Newman

**Fig 4:**
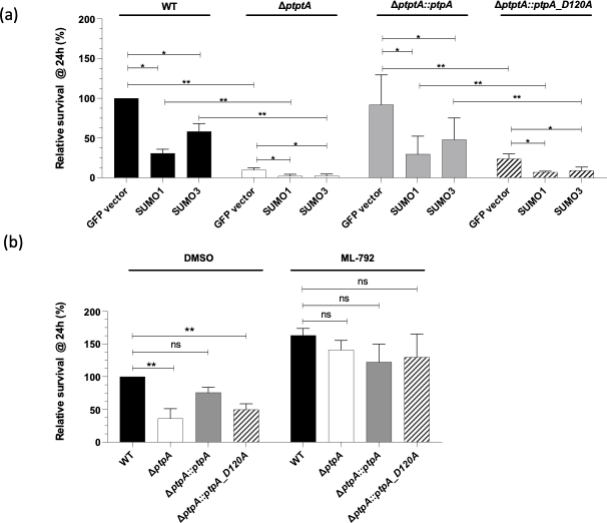
Impact of SUMOylation over-expression or inhibition on intracellular survival of *S. aureus ptpA* strain derivatives. (a) Intracellular survival of *S. aureus* strains in macrophages overexpressing SUMO1 or SUMO2/3 *versus* control macrophages (GFP vector). Intracellular bacteria were counted after cell lysis and relative survival is presented as the ratio of intracellular bacteria at 24-h post-gentamicin compared to cells transfected with an empty GFP-vector, considered as 100%. (b) Macrophages pretreated with ML-792 at 0.5 µM or dimethyl sulfoxide (DMSO) were infected with *S. aureus* strains. Numbers of intracellular bacteria recovered from macrophages at 24-h pGt were counted and are presented as the ratio of intracellular bacteria compared to cells pretreated with DMSO and infected with strain Newman (WT), which were considered as 100%. **P < 0.01; *P < 0.05; and ns: not significant (Mann-Whitney *U* test).

In our next series of experiments, we made use of RAW 264.7 cells overexpressing SUMO1 or SUMO3 moieties to artificially increase the level of SUMOylated proteins in order to substantiate the role of PtpA in the host SUMOylation response to *S. aureus* infection. When these SUMO1 or SUMO3 overexpressing macrophages were challenged with *S. aureus* Newman and the quantity of viable intracellular bacteria was counted after 24-h post-infection, a clear decrease in the intracellular persistence rate of this strain was observed in SUMO1 or SUMO3 overexpressing RAW 264.7 cells, when compared to control macrophages expressing green fluorescent protein (GFP) (Fig. 4a). Notably, a rather similar effect was noticed with the *cis*-complemented **Δ** *ptpA::ptpA* derivative, whereas SUMO1 or SUMO3 overexpressing RAW 264.7 cells challenged with the Δ*ptpA* strain presented considerably lower survival rates. We observed 4.6- and 4-fold reductions between SUMO1 or SUMO3 overexpressing macrophages in comparison to GFP overexpressing macrophages infected with the Δ*ptpA* mutant, while 3.2- and 1.7-fold reductions between SUMO1 or SUMO3 overexpressing macrophages and GFP overexpressing macrophages infected with the WT. A similar trend was also noticed for SUMO1 or SUMO3 overexpressing RAW 264.7 cells infected with the *ptpA_D120A* mutant. These results confirm, as previously observed, that an increase in SUMOylation in host cells has a negative impact on the ability of *S. aureus* to survive inside macrophages (11), and, importantly, demonstrate that PtpA expression is necessary to minimize the SUMOylation host response in order to improve *S. aureus* long-term survival. In addition, the function of PtpA in the regulation of *S. aureus* survival inside macrophages that had been pretreated with an inhibitor of the SUMOylation machinery targeting the SUMO-activating enzyme E1, which is a heterodimer of the SAE1/SAE2 subunits enzyme, was addressed (24). Macrophages that were treated with the inhibitor ML-792 exhibited a substantial increase in the amount of intracellular bacterial cells regardless of the strains used to infect the treated macrophages (Fig. 4b). According to these findings, treatment with the ML-792 inhibitor is able to restore *S. aureus* survival regardless of PtpA.

## DISCUSSION

SUMOylation is an important post-translational modification system deployed by eukaryotes to modulate diverse cellular mechanisms (9). Only lately, researchers started to look at whether pathogenic bacteria might make use of this system for their own benefit, and our understanding of these interactions is still limited (25, 26). Some pathogens interfere with the host SUMOylation machinery (26). The enteropathogenic species *Salmonella* Typhimurium interferes with the SUMOylation host response by inducing the overexpression of two host microRNAs that post-transcriptionally reduce Ubc9 expression (4). *Shigella flexneri* is responsible for the modification of SUMO-conjugated proteins involved in the regulation of mucosal inflammation and epithelial infiltration, respectively (3, 27). Adherent-invasive *Escherichia coli* are thought to restrict autophagy by altering the host’s SUMOylation, thus enabling intracellular proliferation (5). By reducing the induction of host inflammatory pathways, *Klebsiella pneumoniae* diminishes SUMOylation to enhance its infectivity (6). Bacteria such as *Xanthomonas euvesicatoria* (13) and *Yersinia pestis* (12) have been shown to release effectors that are able to imitate host deSUMOylases, which in turn induces deSUMOylation of host proteins. The pore-forming toxin listeriolysin (LLO) produced by *Listeria monocytogenes* was demonstrated to modify the host SUMOylation response by degrading Ubc9 (2). More recently, we demonstrated that *S. aureus* reduces the SUMOylation response in macrophages, thereby promoting its intracellular persistence within this immune cell type (11). However, the bacterial effector(s) that modulate the host SUMOylation response following *S. aureus* ingestion remained unknown. In this study, we show that (i) the secreted protein tyrosine phosphatase PtpA is associated with the reduction of the SUMOylation response in murine macrophages to promote its intracellular persistence, (ii) the PtpA phosphatase function is required for the modulation of the SUMOylation response, (iii) Ubc9 levels are markedly decreased in a PtpA-dependent manner, (iv) the intracellular survival of *S. aureus* Δ*ptpA* cells is significantly decreased in macrophages overexpressing SUMO1 or SUMO3, suggesting the involvement of PtpA in this SUMO-dependent regulation, and (v) when macrophages were treated with the SUMOylation inhibitor ML-792, **Δ** *ptpA* mutants were able to survive inside macrophages to a similar extent as WT cells. Our findings suggest that PtpA is required for a global deSUMOylation in host cells, at least at a later stage of infection (i.e., 24-h pGt), by lowering the level of Ubc9 to promote its intracellular survival. Furthermore, in a previous study, we demonstrated that *S. aureus* PtpA is significantly secreted in macrophages 18 h after infection that could correlate with a late decrease of SUMOylation involving PtpA at 24-h post-infection (21). However, as treatment with the proteasome inhibitor MG132 had no effect on the amount of Ubc9 in RAW 264.7 cells infected with *S. aureus* strain Newman, we assume that the Ubc9 degradation seen in murine macrophages infected with *S. aureus* Newman cells expressing a functional PtpA is independent of the proteasome. Our qRT-PCR studies suggest, furthermore, that PtpA does not markedly affect Ubc9 expression, at least on the transcriptional level. As other virulence effectors released by bacterial pathogens have been demonstrated to downregulate Ubc9 (5), it was already suggested that interfering with host SUMOylation via this critical enzyme of the SUMO machinery is a mechanism utilized by many different kinds of pathogenic bacteria (26), and the same seems to hold truth for *S. aureus*. However, the PtpA-dependent target(s) that is/are responsible for the reduction of Ubc9 seen in *S. aureus*-infected macrophages still remain to be identified, and the specific mechanism of action to be defined. Another limitation of this study is that we tested the impact of *S. aureus* PtpA on host cell SUMOylation only with strain Newman yet, a cytotoxic and mouse pathogenic laboratory strain that features a couple of uncommon characteristics such as the rare *saeS* ^P^ allele leading to a constitutive expression of the *sae* system, and truncations of the fibronectin-binding proteins important for host cell invasion (28 , 29, 30 ). Given that the intracellular survival phenotype of *S. aureus* in professional and non-professional phagocytic cells strongly depends on the host cell type and the infecting bacterial strain (31 , 32, 33), it would be interesting to investigate if different *S. aureus* strains would generate a similar SUMO-response, as well as to test the survival of *S. aureus* in human-derived macrophages such as human peripheral blood-derived monocytic macrophages or THP-1 cells.

Our findings show that a catalytically active form of the PtpA tyrosine phosphatase is required to induce the host SUMOylation reduction; thus, we hypothesize that a Tyr-phosphorylation-dependent control mechanism may be involved. As the *ptpA* deletion mutant displayed no clear effect on *ubc9* transcription, post-translational regulation seems to be the most likely mode of regulation. One possible mechanism might be that PtpA affects the phosphorylation status of Ubc9, which in turn influences the stability of the protein (34). However, due to the fact that PtpA is a tyrosine phosphatase and that Ubc9 is known to be phosphorylated on threonine residues (35, 36), it is unlikely that PtpA directly dephosphorylates Ubc9, but might influence the phosphorylation status of Ubc9 by a cascade of kinases that are phosphorylated on tyrosine residues. One putative candidate for such a scenario is the Ser/Thr protein kinase Akt that is activated through tyrosine phosphorylation (37), and directly phosphorylates Ubc9 at Thr35, favoring the SUMO-charged form of Ubc9 (35). Another candidate would be cyclin-dependent kinase 1 (Cdk1, also known as cell division cycle 2 [Cdc2]), which in cooperation with cyclin B fosters Ubc9 phosphorylation at Ser71 to enhance Ubc9 stability and SUMOylation activity (34, 36, 38). As Cdk1 activity itself is regulated by protein tyrosine and threonine phosphorylation (reviewed in reference 39), one may speculate that PtpA might affect Ubc9 stability via Cdk1 dephosphorylation at Tyr15.

Another potential candidate by which PtpA might affect SUMOylation is SUMO-specific protease 1 (SENP1), which acts as an endopeptidase to generate mature SUMO for protein conjugation and as an isopeptidase to remove conjugated SUMO from targets (40). SENP1 was recently identified as a substrate of protein tyrosine kinase Lck, which phosphorylates SENP1 at Tyr270, thereby rendering its endopeptidase and isopeptidase activities (41). Lck was originally described as lymphocyte-specific kinase but was also found in RAW 264.7 cells (42).

Predictably, low levels of Ubc9 are accompanied by decreased amounts of SUMOylated proteins. In this context, one would expect to observe an accumulation of unconjugated SUMO moieties. However, this was not the case in our Western blot analyses, as the fast-migrating band (~20 kDa) reactive to the anti-SUMO antibodies was always weak to undetectable in our immunoblots of *S. aureus* Newman-infected cells, and on a comparable level to the corresponding signals seen on immunoblots performed with cell lysates of uninfected RAW 264.7 cells, or of those infected with the PtpA-defective strains (data not shown). These observations suggest that *S. aureus* PtpA might also interfere with host cell SUMOylation by affecting the availability of SUMO, potentially via interference with the Lin-28/let-7 pathway (43).

In conclusion, the current work shows for the first time that the secreted phosphatase PtpA is capable of reducing the Ubc9 conjugation enzyme level to impede host SUMOylation response in *S. aureus* Newman infected murine macrophages, thus promoting the intracellular survival of the ingested bacterial cells in this immune cell type. SUMOylation crosstalk during bacterial infection represents a promising area of research that will not only enhance our knowledge of how SUMOylation occurs in cells but may also reveal potential targets for therapeutic treatment against *S. aureus* infections and persistence at long-term infections.

## MATERIALS AND METHODS

### Bacterial strains and growth conditions

Strains and plasmids used in this study are listed in Table 1. Sequencing was used to confirm all mutant strains and plasmids used for this study. Strains of *Escherichia coli* were cultivated at 37°C in Luria-Bertani (LB) medium with the addition of 100 mg/mL ampicillin when needed. *S. aureus* isolates were either cultured in TSB (Becton Dickinson) at 37°C and 225 rpm with a culture to flask volume of 1:10, or plated on tryptic soy agar (TSA; Becton Dickinson) supplemented with 10 mg/mL erythromycin when required. Bacterial growth in 96-well plates was observed using a microplate reader (Tecan, Lyon, France).

**TABLE 1:** Strains and plasmids used in this study

Strain	Description^*a*^	Reference or source
*S. aureus*		
Newman	Laboratory strain, wild type	
Newman Δ*ptpA*	Newman Δ*ptpA*::lox66-*aphA*III-lox71; Kan^R^	(22)
Newman Δ*ptpA*::*ptpA*	Newman Δ*ptpA* derivative *cis*-complemented with pEC1_*ptpA-*Flag; Erm^R^	(22)
Newman Δ*ptpA*::*ptpA_D120A*	Newman Δ*ptpA* derivative *cis*-complemented with pEC1_*ptpA_D120A*-Flag; Erm^R^	This study
RN4220 Δ*ptpA*	RN4220Δ*ptpA::lox72*	(22)
RN4220 Δ*ptpA*_lox_*aph*	RN4220 Δ*ptpA*::lox66-*aphA*III-lox71	(22)
*E. coli*		
IM08B	*E. coli* DC10B derivative harboring *hsdS* of *S. aureus* strain NRS384, Δ*dcm*	(44)
TOP10	*E. coli* derivative ultra-competent cells used for general cloning	Invitrogen
Plasmids		
pEC1	pUC19 derivative containing the 1.45 kb *Cla*l *erm*(B) fragment of Tn*551*	(45)
pEC1_*ptpA_D120A-*Flag	pEC1 with a 1.4 kb fragment covering the *ptpA* ORF including a C-terminal flag tag and the aspartate 120 mutated to alanine, and 0.7 kb of the upstream region	This study

^*a*^ Erm^R^, erythromycin resistant; Kan^R^, kanamycin resistant; ORF, open reading frame.

### Construction of the *S. aureus* ptpA *cis*-complementation strain Newman ptpA::ptpA_D120A

For *cis*-complementation of the *ptpA* mutation in strain Newman Δ*ptpA* with a *ptpA* derivative harboring the D120A exchange, the vector pEC1_*ptpA* (22) was used as a template to generate the suicide plasmid pEC1_*ptpA_D120A* by using the QuikChange Site-Directed Mutagenesis kit (Agilent Technologies) with the primer #1559 (5′-GGAAGAGAGTGATGTACCAGCTCCATACTACACGAATAATT-3′). Plasmid pEC1_*ptpA_D120A* was then electroporated into the strain RN4220 Δ*ptp,* a marker-free Δ*ptpA* variant of *S. aureus* strain RN4220, which was previously constructed (22). The RN4220 derivative that integrated pEC1_*ptpA_D120A* was subsequently used as a donor for phage transducing the *cis*-integrated pEC1_*ptpA_D120A* genome region into Newman Δ*ptpA* (22), thereby replacing the *aphAIII*-tagged *ptpA* deletion with the *ptpA_D120A* derivative. Replacement of the *ptpA* deletion with *ptpA_D120A* in Newman Δ*ptpA::ptpA_D120A* was confirmed by sequencing.

### Macrophages culture and infection

The murine macrophage cell line RAW 264.7 (mouse leukemic monocyte macrophage, ATCC TIB-71) was cultured in Dulbecco’s modified Eagle’s medium (DMEM) (ThermoFisher Scientific), which was augmented with 10% fetal bovine serum and kept at 37°C in a humidified atmosphere containing 5% carbon dioxide. Lentiviral-transduced RAW 264.7 cell lines that expressed 6His-tagged SUMO1 and SUMO3 proteins were previously generated (11). RAW 264.7 cells (5 × 10^5^ cells/mL in 24 well plates) were challenged with *S. aureus* at an MOI of 20:1 (bacteria:cells), and the cell mixtures were then incubated for 1 h at 37°C and 5% CO_2_. Residual extracellular bacteria were eliminated by incubating the RAW 264.7 cells with gentamicin (100 µg/mL) for 30 min after the cells had been washed once with phosphate-buffered saline (PBS). Following gentamicin treatment, macrophages were washed twice with PBS (T0), and subsequently incubated for 24 h in DMEM in the presence of 5 µg/mL lysostaphin (Ambi Products LLC, USA). Afterward, macrophages were lysed by 0.1% Triton X100 treatment (T24), and serial dilutions of the lysates were plated on TSA plates, which were incubated for 24 h at 37°C. The number of bacterial colonies at T24/number of bacterial colonies at T0 × 100% was used to calculate the survival rate of bacteria.

### Immunoblotting

Infected macrophages were lysed in 100 µL of 2.5× Laemmli buffer, boiled for 10 min at 95°C, sonicated for 10 s at 50% amplitude of a 20 kHz sonifier (Digital, Model 450-D, Branson), and centrifuged for 1 min at 12,000 × *g*. Proteins were separated on SDS-PAGEs, transferred to polyvinylidene difluoride (PVDF) membranes, and analyzed by Western blotting using an anti-SUMO1 (#21C7, Developmental Studies Hybridoma Bank) or anti-SUMO2/3 antibody (#8A2, Developmental Studies Hybridoma Bank) as primary antibody, and an HRP-coupled donkey-anti-mouse antibody as secondary antibody (Jackson ImmunoResearch, Interchim, France). The immunoblots were detected with the Enhanced Chemiluminescence Detection kit (ChemiDoc, BioRad) and quantified using Image Lab software (BioRad).

### qRT-PCR

Total RNAs were extracted using the RNeasy plus Mini kit (Qiagen, GmbH, Germany) following the manufacturer’s instructions. To measure the levels of mRNA expression, 1 µg of total RNA was reverse-transcribed using the SuperScript III Reverse Transcriptase kit from Invitrogen. Using SYBR Green qPCR Master Mix (Roche) and specific primers (Table 2), qRT-PCR was carried out using a LightCycler 480 (Roche, France). As internal controls for mRNA quantification, the mouse β-actin gene was utilized. Using the Ct technique, the fold-induction was determined as follows: ∆∆Ct = (Ct target gene − Ct internal control) treatment − (Ct target gene − Ct internal control) non-treatment, and the final data were derived from 2 − ∆∆Ct.

**TABLE 2:** qRT-PCR primers used in this study

Gene target	Primer	Sequence (5′−3′)
*β-actin*	Forward	AGCCATGTACGTAGCCATCC
	Reverse	CTCTCAGCTGTGGTGGTGAA
*ubc9*	Forward	CCTCAGCCGCCTTGCGCAGGA
	Reverse	ACTGTGCCAGAAGGATACACG

### Statistical analyses

The statistical significance of changes between groups was determined using the GraphPad software package Prism 9.4.0. *P* values <0.05 were considered statistically significant.
